# Pulmonary findings in COVID-19: radiology correlates with the histopathological post-mortem findings in patients with fatal acute disease

**DOI:** 10.1177/02841851261425663

**Published:** 2026-03-25

**Authors:** Ann Mari Svensson, Laszlo Szekely, Hans Brunnström, Anna Kistner, Cristian Ortiz-Villalón

**Affiliations:** 1 Department of Radiology, Karolinska University Hospital, Stockholm, Sweden; 2 Department of Molecular Medicine and Surgery, Karolinska Institutet, Stockholm, Sweden; 3 Department of Clinical Pathology and Cancer Diagnostic, 27106 Karolinska University Hospital, Stockholm, Sweden; 4 Department of Oncology-Pathology, Karolinska Institutet, Stockholm, Sweden; 5 Department of Genetics, Pathology, and Molecular Diagnostics, Lund University Hospital, Lund, Sweden; 6 Department of Clinical Sciences, Division of Pathology, Lund University, Lund, Sweden; 7 Department of Nuclear Medicine and Medical Physics, Karolinska University Hospital, Stockholm, Sweden; 8 Department of Nuclear Medicine and Medical Physics, Theranostics Trial Center, Karolinska University Hospital, Stockholm, Sweden; 9 Section for Pathology, Center for Laboratory Medicine, Østfold Hospital Trust, Sarpsborg, Norway

**Keywords:** Acute respiratory distress syndrome, diffuse alveolar damage, organizing pneumonia, chest computed tomography, lung biopsy

## Abstract

**Background:**

Progressive respiratory failure is the leading cause of death in patients with severe COVID-19. Histopathological findings in acute severe COVID-19 are foremost based on post-mortem findings. On computed tomography (CT), acute COVID-19 pneumonia is characterized by ground-glass opacities (GGOs) and, later, by a crazy-paving pattern (CPP) and consolidations.

**Purpose:**

To investigate if CT patterns corresponded to histopathological post-mortem findings.

**Material and Methods:**

Eight patients were identified with a chest CT performed between testing positive for COVID-19 and death. CT images, histological slides, and medical records were retrospectively reviewed. The lungs were photographed during the gross investigation to ascertain the exact position of the tissue blocks in relation to the relevant anatomical structures. Each slide was compared side by side with the in vivo chest CT pattern on the corresponding site.

**Results:**

At CT, the most predominant finding was GGOs, present in all eight cases. CPP was observed in 6/8 (75%) patients, and consolidation in 7/8 (87.5%) patients, both predominantly located in the lower lung zones. In 5 (62.5%) patients, so-called fibrotic-like changes were present. In four patients with CT angiogram, no findings of pulmonary thromboembolism were present. At autopsy, all patients demonstrated macroscopic consolidation, while pleural effusion was seen in 2 (25%) cases. Microscopically, edema was present in all cases, hyaline membranes in 7/8 (87.5%) cases, but no signs of acute interstitial inflammation were observed. Thromboembolic findings were evident in 4 (50%) patients, of whom two were negative on CT and 3 (37.5%) cases had fibrosis.

**Conclusion:**

The results demonstrate a clear association between radiological signs of GGO, consolidation and features of organizing pneumonia to microscopical signs of edema and diffuse alveolar damage. However, fibrotic-like changes and thromboembolism in the small vessels had a poorer compliance.

## Introduction

The SARS-CoV-2 virus significantly impacts the lungs, and progressive respiratory failure is the leading cause of death in patients with severe COVID-19 (1). Computed tomography (CT) has been a valuable tool in addition to reverse transcription polymerase chain reaction (RT-PCR) in the diagnosis of COVID-19 (2,3) due to its characteristic spatiotemporal findings on CT (4). On CT, acute COVID-19 pneumonia is characterized by bilateral peripheral ground-glass opacities (GGOs), followed by perilobular opacities or, occasionally, a reversed halo sign and, in severe cases, by a crazy-paving pattern (CPP) and consolidations (4).

GGOs appear as hazy areas of increased lung density, representing partial filling of the alveoli. The perilobular opacities and reversed halo sign both indicate an underlying organizing pneumonia (OP) injury pattern. Consolidations manifest as areas of complete airspace filling, indicating more severe involvement of the lung parenchyma. The CPP pattern refers to a linear pattern (interlobular septal thickening) being superimposed on GGOs, which can be seen in the advanced stages of various lung diseases and represents diffuse alveolar damage (DAD) (5). Both DAD and OP represent pathological patterns of lung injury and are the lung's response to an insult (6,7). Reticulations associated with bronchial dilatation and distortion in areas of consolidation or GGO are believed to be an interim finding during the course of OP for which the term “fibrotic-like changes” has been proposed (8). It could be a precursor of fibrosis but should not be regarded as manifest fibrosis (8).

Knowledge about histopathological findings in acute severe COVID-19 is essentially based on post-mortem findings (9,10). In COVID-19 patients developing acute respiratory distress syndrome (ARDS), the lungs, at macroscopic evaluation, typically show consolidations with edema and mucus in the airways as well as hemorrhage in the lung parenchyma and, in some cases, the presence of foremost peripheral thromboembolism and serous pleural effusion (11–13).

Microscopically, alveolar edema and fibrin precipitation occur in acute lung injury. If hyaline membranes, remnants of necrotic epithelium and endothelium, as well as fibrin, arise, the condition is defined as DAD, which is reported in most cases of fatal COVID-19 (14). Patchy chronic inflammation (lymphocytes and monocytes/macrophages), desquamated epithelium, reactive type II pneumocytes, and squamous cell metaplasia are common additional findings. Focal intraparenchymal bleeding has been reported in some cases, as well as circulatory stasis and fibrin thrombi in arterioles (13,15,16). Focal minimal or pronounced neutrophilic infiltrates have been reported (13,17).

**Fig. 1.**
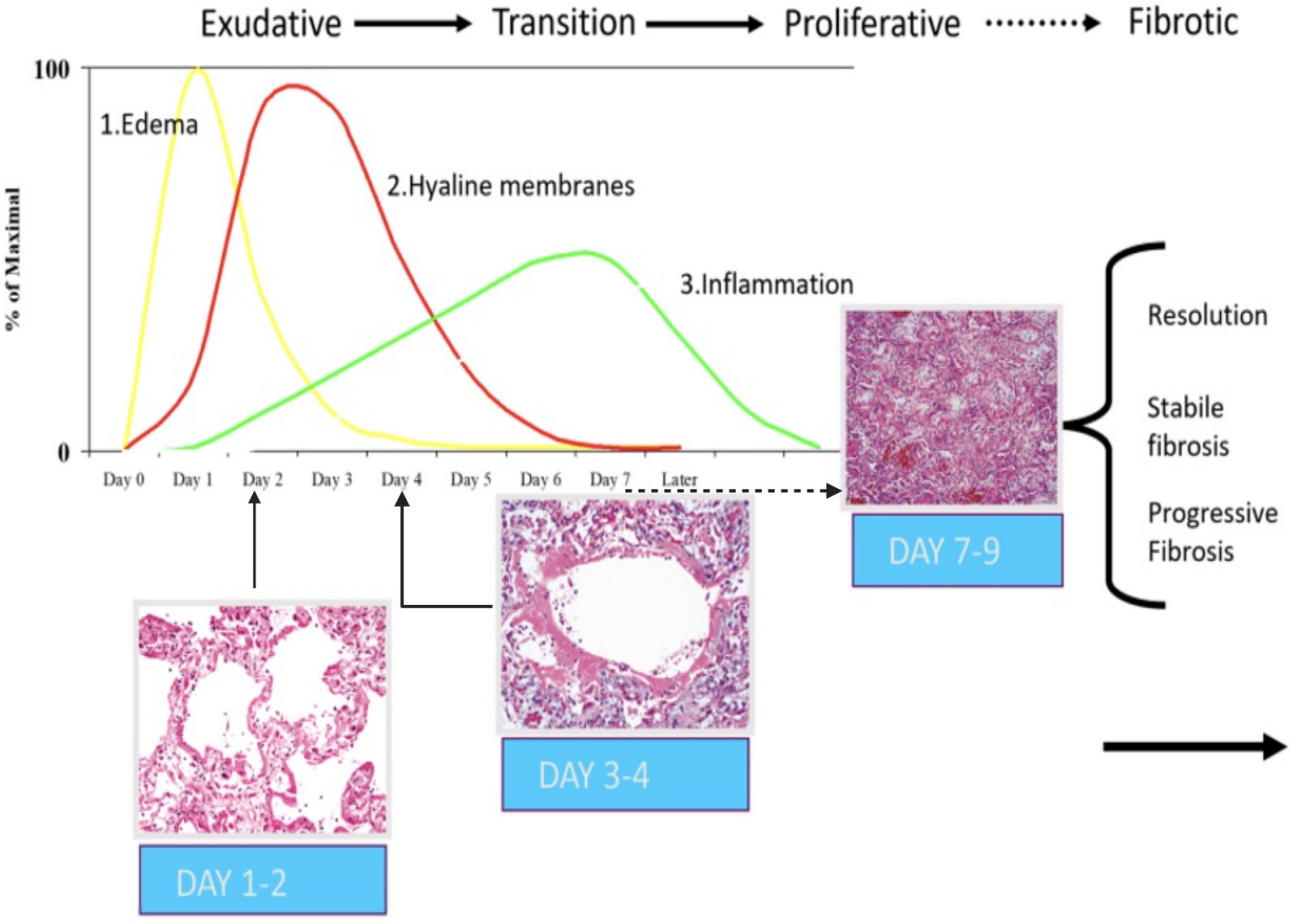
Phases of ARDS and representative histological images of diffuse alveolar damage (DAD). Days 1–2 (exudative phase): Edema in the interalveolar space, dilated capillaries, and interstitial edema, with subsequent development of intra-alveolar edema. Days 3–4 (transition phase): formation of hyaline membranes by injured pneumocytes, along with fibrin aggregates, obstruction of the alveolar lumen, and cellular debris. Days 7–9 (proliferative phase): proliferation of type II pneumocytes and migration of fibroblasts lead to organization of the intra-alveolar exudate, with progressive reduction of alveolar spaces. ARDS, acute respiratory distress syndrome; DAD, diffuse alveolar damage.

During the transition from exudative (acute) to proliferative (reparative/organizing) phase, loose fibrosis appears. In the proliferative phase, the edema has typically disappeared, and hyaline membranes—and later inflammation—also vanish gradually. Fig. 1 illustrates the temporal evolution of DAD.

The aim of the present study was to investigate whether the CT patterns found during the progression of COVID-19 disease corresponded to the histopathological post-mortem findings, and whether the anatomical localization of individual tissue blocks could retrospectively be matched to corresponding regions on CT. If reliable retrospective localization can be achieved, this approach may facilitate larger-scale studies integrating imaging and histopathology.

## Material and Methods

### Methods

The study was approved by the ethical committee, numbers 2020-02446, 2020-01882, 2021-04735.

In this study, we retrospectively included all patients with COVID-19 symptoms who tested positive for SARS-CoV-2, underwent chest CT between diagnosis and death, and were autopsied between 1 March 2020 and 31 August 2021. Patient data, including CT scans, medical records, and histological slides, were reviewed.

Patient records provided information on symptom onset, CT scan timing, mechanical ventilation duration, causes of death, known COVID-19 risk factors, body mass index (BMI), co-morbidities, and complications like pneumothorax.

#### CT imaging in relation to time of death

CT scans were closely linked to the time of death, with three patients having a scan within 6 h of death, and one scanned immediately after death. Three patients underwent multiple CT scans before death (two had two scans and one had three scans).

#### CT imaging examination

CT scans were examined by a radiologist with 13 years of thoracic experience, blinded to the clinical and autopsy details. Key radiological findings, such as GGOs, airspace consolidations, fibrotic changes, pleural effusions, and pneumothorax, were recorded. Pulmonary involvement was classified into four categories based on parenchymal involvement (0–25%, >25–50%, >50–75%, >75–100%). Lesions affecting more than 50% were considered widespread parenchymal abnormalities (WPAs) (18).

#### Histopathology examination

Autopsy material was examined by two pathologists. The organs had previously been photographed during the gross investigation to ascertain the exact position of the tissue blocks in relation to the relevant anatomical structures. Samples from relevant organs were fixed in 10% neutral-buffered formalin (corresponding to 4% formaldehyde) and embedded in paraffin blocks. Sections with a thickness of 5 µm were cut and stained with hematoxylin and eosin in an automated device. The number of tissue blocks from lungs per case was in the range of 5–12.

Histopathological findings; such as DAD/hyaline membranes, consolidation, pleural effusion, and thromboembolism, were recorded. Pleural effusions under 30 mL and post-mortem blood presence were considered artifacts and excluded.

#### Correlation between histopathology and thoracic CT imaging

For the correlation between CT and histopathology, tissue blocks were matched with corresponding areas on CT scans. This matching was done through joint sessions between radiologists and pathologists, using the lung's anatomical lobes for guidance (RUL, RLL, RML, LUL, LLL) (19). Tissue blocks, measuring 2 × 1 cm, were correlated with CT regions, considering surrounding lung parenchyma for more reliable pattern recognition. The correlation was qualitative, based on visual and anatomical concordance between histological sections and CT findings. Only cases in which anatomical localization could be established with confidence were recorded. Except for one case with extensive pneumothorax and lung collapse, matching was successful in all other cases based on available anatomical documentation.

## Results

A total of 36 patients with symptoms compatible with COVID-19 and a positive SARS-CoV-2 test underwent autopsy at our hospital during the study period. Of these, eight patients had undergone chest CT between the time of testing positive and death. None of the eight patients had been vaccinated against COVID-19.

In addition to the included cases, three other patients had undergone chest imaging (chest X-ray) but not thoracic CT scan and were therefore not eligible for inclusion.

### Clinical features

Patient demographics are summarized in Table 1.

**Table 1.** Demographics, risk factors, and clinical course in relation to CT in fatal SARS-CoV-2 infection.

Case no.	Age (years)	Sex	Immediate cause of death	Days between clinical onset and death	Days between clinical onset and CT scan(s)	Days between last CT scan and death	Days on mechanical ventilation	BMI (kg/m^2^)	Risk factors for severe COVID-19	Pneumothorax during hospitalization	Other complications during hospitalization	Other clinically significant conditions
1	56	F	ARDS	21	11, 21	0	7	33	Obesity	N	Acute renal failure	
2	69	M	ARDS	4	4	0	0	29	HTN	Y	None	
3	77	F	Pulmonary embolism	18	11	7	0	30	Obesity	N	None	
4	61	M	ARDS	56	9, 27, 39	17	41	25	Diabetes mellitus type II	Y	Staph. aureus sepsis	
5	72	F	ARDS	18	6, 18	0	10	26	Lung transplanted (f-ILD), HTN	Y	Candida sepsis	Lung transplanted
6	39	F	ARDS	22	13	9	2	36	Obesity, hypogammaglobulinemia	N	Pneumonia, preeclampsia	Pregnancy, GPA
7	66	M	ARDS	14	7	7	0	31	Obesity	N	None	
8	64	M	ARDS	30	20	10	6	28	HTN	N	Acute renal failure, VAP	

Clinical, radiological, and pathological baseline characteristics of eight patients who died after SARS-CoV-2 infection. Data include demographic features, disease course, co-morbidities, ventilatory support, and complications. Time intervals are reported in days unless otherwise specified.

ARDS, acute respiratory distress syndrome; BMI, body mass index; CT, computed tomography; F, female; f-ILD, fibrotic interstitial lung disease; GPA, granulomatosis with polyangiitis; HTN, hypertension; M, male; Staph, Staphylococcus; VAP, ventilator-associated pneumonia.

Eight patients (4 men, 4 women; median age = 66 years; age rang e= 39–78 years) were included in the study. In 7/8 (87.5%) patients, the cause of death was ARDS; the remaining patient (12.5%) died from pulmonary embolism. The median time between clinical onset and death was 20 days (range = 4–56 days).

A majority (75%) of the patients were on mechanical ventilation or had been on mechanical ventilation before death, with a median of 5 days of intubation. One additional patient had mechanical ventilation for only a couple of hours before death and was classified as non-mechanical ventilation, assuming no histological signs of barotrauma were likely to have developed in this brief time span.

Apart from male sex, 6 (75%) patients had one known risk factor, while the other 2 (25%) patients each had two risk factors for severe COVID-19, most commonly high BMI or hypertension (Table 1). During hospitalization, 5 (62.5%) patients had complications that may have contributed to the outcome, including sepsis, superimposed bacterial- or ventilator-associated pneumonia (VAP), or acute renal failure (Table 1).

### Radiological findings

Radiological findings are presented in Table 2. Median time between the last CT scan and death was 7 days (range = 0–17 days). None of the eight patients demonstrated normal attenuation on any of their chest CT examinations*.* In 7 (87.5%) patients, more than 50% of the parenchyma was affected, i.e. WPA. The most predominant finding was GGOs, which were present in all eight cases. CPP was present in 6 (75%) patients and consolidation in 7 (87.5%) patients, both predominantly in the lower lung zones. All patients exhibited a perilobular pattern, which was present focally in five cases and constituted the predominant finding in 3 (37.5%) patients. No reversed halo sign was found. In 5 (62.5%) patients, so-called fibrotic-like changes were present. Four (50%) CT scans were performed as a CT pulmonary angiogram (CTPA), on which there were no findings of pulmonary thromboembolism.

**Table 2.** Radiologic patterns: CT findings in fatal SARS-CoV-2 infections.

Case no.	CT on day x from symptom starts	Days before death	GGOs	Perilobular pattern	Consolidations	Crazy paving	Fibrotic like changes*	Honeycombing	% affected lung	Reverse halo sign	Thromboembolism	Pneumothorax
1	21	0	Y	Y	Y^†^	Y	Y	N	50–75	N	N	N
2	4	0	Y	Y	Y^†^	Y	Not assessable^‡^	NO	>50^‡^	Not assessable^‡^	Not assessable^‡^	Y
3	11	7	Y^†^	Y^†^	N	N	Y	N	50–75	N	N	N
4	39	17	Y^†^	Y	Y^†^	Y	Y	N	75–100	N	Not assessable^‡^	Y
5	18	0	Y^†^	Y	Y	Y^†^	YES	NO	75–100	N	Not assessable^‡^	Y
6	13	9	Y	Y^†^	Y^†^	N	N	N	25–50	N	N	N
7	7	7	Y	Y^†^	Y^†^	Y	N	N	50–75	N	N	N
8	20	10	Y^†^	Y	Y	Y^†^	Y	N	50–75^§^	N	Not assessable^‡^	N

Radiologic summary of eight deceased patients with confirmed SARS-CoV-2 infection, based on the last CT performed before death. Time intervals (in days) are reported from symptom onset to CT and from CT to death. Patterns were defined according to Fleischner Society guidance, and dominant parenchymal patterns were categorized by visual assessment and indicated by a dagger (^†^).

*Fibrotic-like changes were defined as reticulation with associated bronchial dilatation and distortion within areas of consolidation or GGOs.

† Dominant radiological finding,

‡ Not assessable: patients 2, 4, 5, and 8 underwent CT without contrast. Patient 2 also had lung collapse precluding precise assessment,

§ CT of the abdomen was performed; lung coverage extended from the carina caudally, excluding the apices.

CT, computed tomography; GGO, ground-glass opacity.

### Pattern evolution in patients with multiple CT scans

**Fig. 2.**
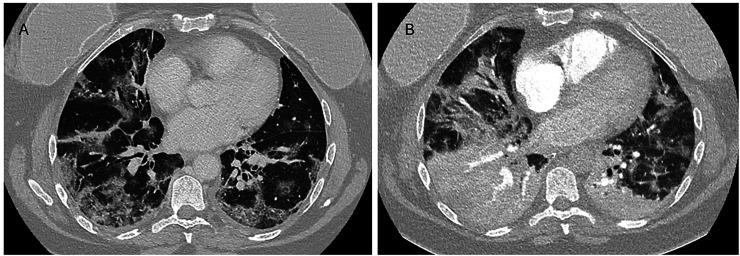
Computed tomography images from patient 1. (a) Day 11 after clinical onset. (b) Day 21 after clinical onset (day of death).

In the three patients (1, 4 and 5) who underwent multiple chest CT scans during hospitalization, the following assessment of temporal changes could be made:

Patient 1’s initial scan showed perilobular opacities with reticulations and CPP. The follow-up scan demonstrated progression to dense lobar consolidation and new hypoattenuating areas suggestive of necrosis or infarction (Fig. 2).

**Fig. 3.**
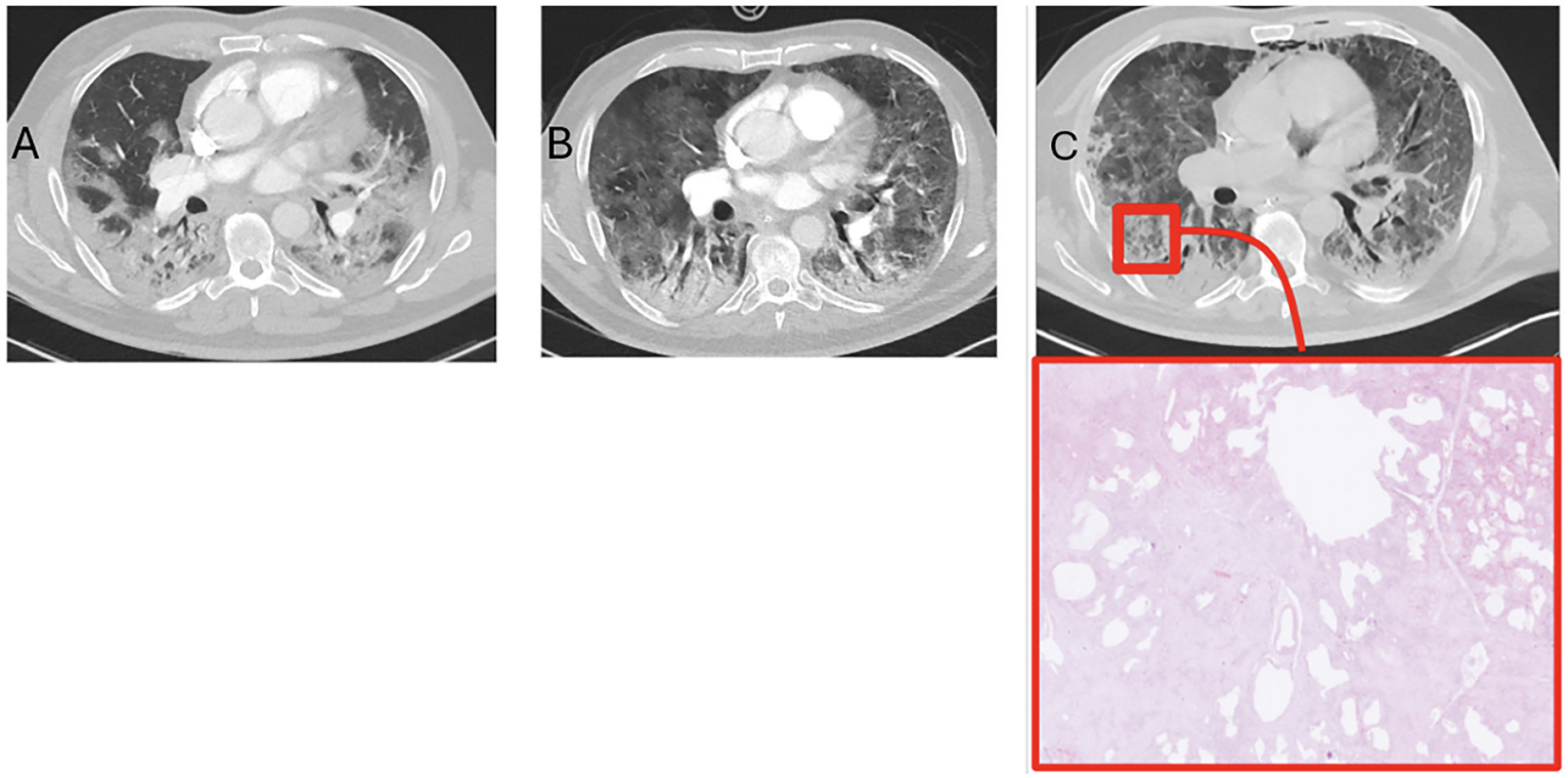
Computed tomography images from patient 4. (a) Day 9 after clinical onset. (b) Day 27 after clinical onset. (c) Day 39 after clinical onset (17 days before death) – bottom row shows a histopathological image from a post-mortem biopsy on day 55 from the corresponding anatomical location (see also Fig. 5 for full histological details).

In patient 4, initial consolidations partially regressed on follow-up, while new GGOs appeared in previously unaffected areas. The last scan showed increased interlobular septal thickening and intralobular lines, denser ground glass, and signs of barotrauma (Fig. 3).

**Fig. 4.**
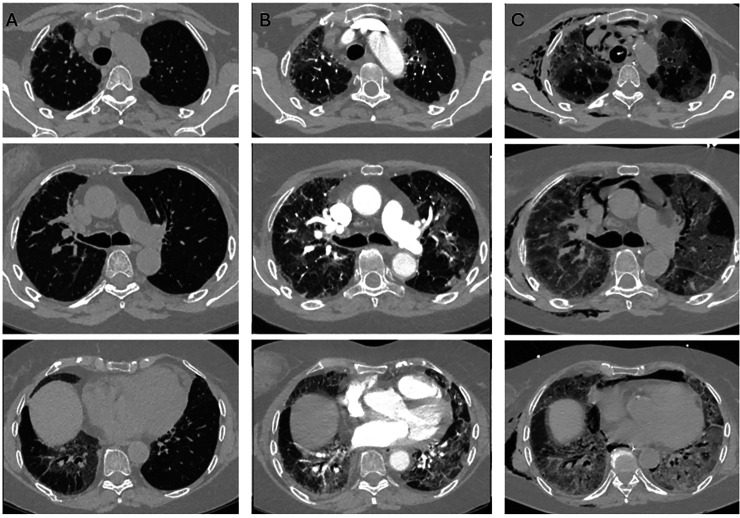
Computed tomography images from patient 5. (a) Baseline scan before COVID-19, performed for follow-up of pre-existing fibrosis. The patient had previously undergone unilateral lung transplantation due to fibrotic NSIP. (b) Day 6 after clinical onset: bilateral GGOs, early reticulations, and focal areas of OP. (c) Day 18 after clinical onset (day of death): marked progression of GGOs. Reticulations and bronchial dilatation had increased in both lungs, with widespread crazy-paving pattern. Notably, no discernible difference in pattern or extent was observed between the transplanted and native lungs, and the caliber pre-existing traction bronchiectasis in the native lung remained unchanged. GGO, ground-glass opacity; NSIP, non-specific interstitial pneumonia; OP, organizing pneumonia.

In patient 5, GGOs and early reticulations progressed to widespread CPP. Pre-existing traction bronchiectasis remained unchanged, but new fibrotic-like changes appeared (Fig. 4).

### Pathological findings

The findings from the autopsies are found in Table 3. All eight patients showed macroscopic consolidation, with the presence of pleural effusions in 5 (62.5%) cases. Microscopically, edema was present in all cases, and hyaline membranes were present in 7 (87.5%) cases.

**Table 3.** Post-mortem pulmonary gross and histologic findings in SARS-CoV-2 infection.

Case no.	Weight (g)	Consolidations	Pleural effusion	Edema	DAD/hyaline membranes	Alveolar neutrophile infiltrate	Organizing pneumonia	Histiocytic giant cells	Thromboembolism small vessels	Thromboembolism pulmonary artery	Vasculitis	Hemorrhage	Squamous metaplasia	Hyperbar artefact	Fibrosis
1	2570	Y	N	Y	Y	N	N	Y	Y	N	N	Y	Y	Y	N
2	1227	Y	Y	Y	Y/AFOP	N	Y	Y	Y	N	N	Y	Y	N	N
3	1587	Y	Y	Y	N	N	Y	Y	Y	Y	N	N	N	N	N
4	2217	Y	N	Y	Y	N	Y	Y	N	N	N	N	Y	Y	Y
5	1709	Y	N	Y	Y	Y	Y	Y	N	N	N	N	Y	N	N
6	1346	Y	Y	Y	Y	N	Y	Y	N	N	N	N	N	N	Y
7	1743	Y	Y	Y	Y	N	Y	Y	N	N	N	Y	Y	N	Y
8	3654	Y	Y*	Y	Y	N	Y	Y	Y	N	N	Y	Y	N	N

Lung tissue samples were obtained post-mortem and assessed for macroscopic and microscopic features of acute and organizing lung injury. The presence or absence of each finding is reported for all patients. Organizing pneumonia was defined by the presence of intra-alveolar fibroblastic tissue (Masson bodies), and DAD was identified by hyaline membranes and alveolar collapse.

DAD, diffuse alveolar damage; OP, organizing pneumonia.

Neutrophilic inflammation was observed in 1 (12.5%) patient, but it was confined to the alveolar spaces and did not involve the interstitium. Fibrin deposition in the alveolar spaces forming fibrin plugs, consistent with OP, was seen in 6 (75%) cases. Pronounced reactive hyperplasia of type II pneumocytes and the presence of multinucleated giant cells were noted in all cases. Thromboembolic findings were evident in 4 (50%) patients, and fibrosis in 3 (37.5%) cases (Table 3).

### Radiological-pathological association

Radiologic–pathologic pattern agreement across anatomically matched lung regions is presented in Table 4.

**Table 4.** Radiologic–pathologic pattern agreement across anatomically matched lung regions in fatal SARS-CoV-2 infections.

Case no.	No. of anatomically matched blocks	Agreement: edema, inflammation, GGO	Agreement: consolidation	Agreement: signs of OP	Agreement: signs of DAD	Agreement: fibrotic like changes/loose fibrosis	Agreement thromboembolism pulmonary artery
1	4	Y	Y	N	Y	N	Y
2	1	Y	Y	Not assessable	Not assessable	Not assessable	Not assessable
3	2	Y	Y	Y	Y	N	N
4	3	Y	Y	Y	Y	Y	Not assessable
5	3	Y	Y	Y	Y	Not assessable	Not assessable
6	4	Y	Y	Y	N	N	Y
7	5	Y	Y	Y	Y	N	Y
8	3	Y	Y	Y	Y	N	Not assessable

Pattern agreement was assessed by correlating histopathological findings from anatomically localized tissue samples with CT appearances at the corresponding sites. Agreement was recorded as “Yes” when similar patterns were identified on both CT and histopathology or “No” if the patterns differed. Agreement was considered present if the following radiologic–pathologic correlations were observed: edema/inflammation on histopathology and GGO on radiology. Signs of OP: radiology = perilobular pattern; histopathology = OP. Signs of DAD: radiology = crazy paving; histopathology = DAD. Fibrotic-like changes were defined as reticulation with associated bronchial dilatation and distortion within areas of consolidation or GGO and corresponding pattern on histopathology loose fibrosis. “Not assessable” indicates that radiologic interpretation was not possible due to technical limitations, such as lung collapse in patient 2 and non-contrast CT in patients 2, 4, 5, and 8.

CT, computed tomography; DAD, diffuse alveolar damage; GGO, ground-glass opacity; OP, organizing pneumonia.

Among the matched samples, most radiological findings correlated well with specific lung histological changes. GGOs, consolidation, signs of OP, and signs of DAD, all demonstrated topographic concordance, whereas thromboembolic findings less, and fibrotic-like changes only in one case.

Only one case did not show radiological-pathological association for signs of OP (patient 1, zero days between last CT scan and death) and one did not show an association for signs of DAD (patient 6, 9 days between last CT scan and death).

All included patients demonstrated GGOs and all but one (patient 3, who died from pulmonary embolism) also exhibited CPP or consolidations on CT, in combination with histological evidence of edema.

**Fig. 5.**
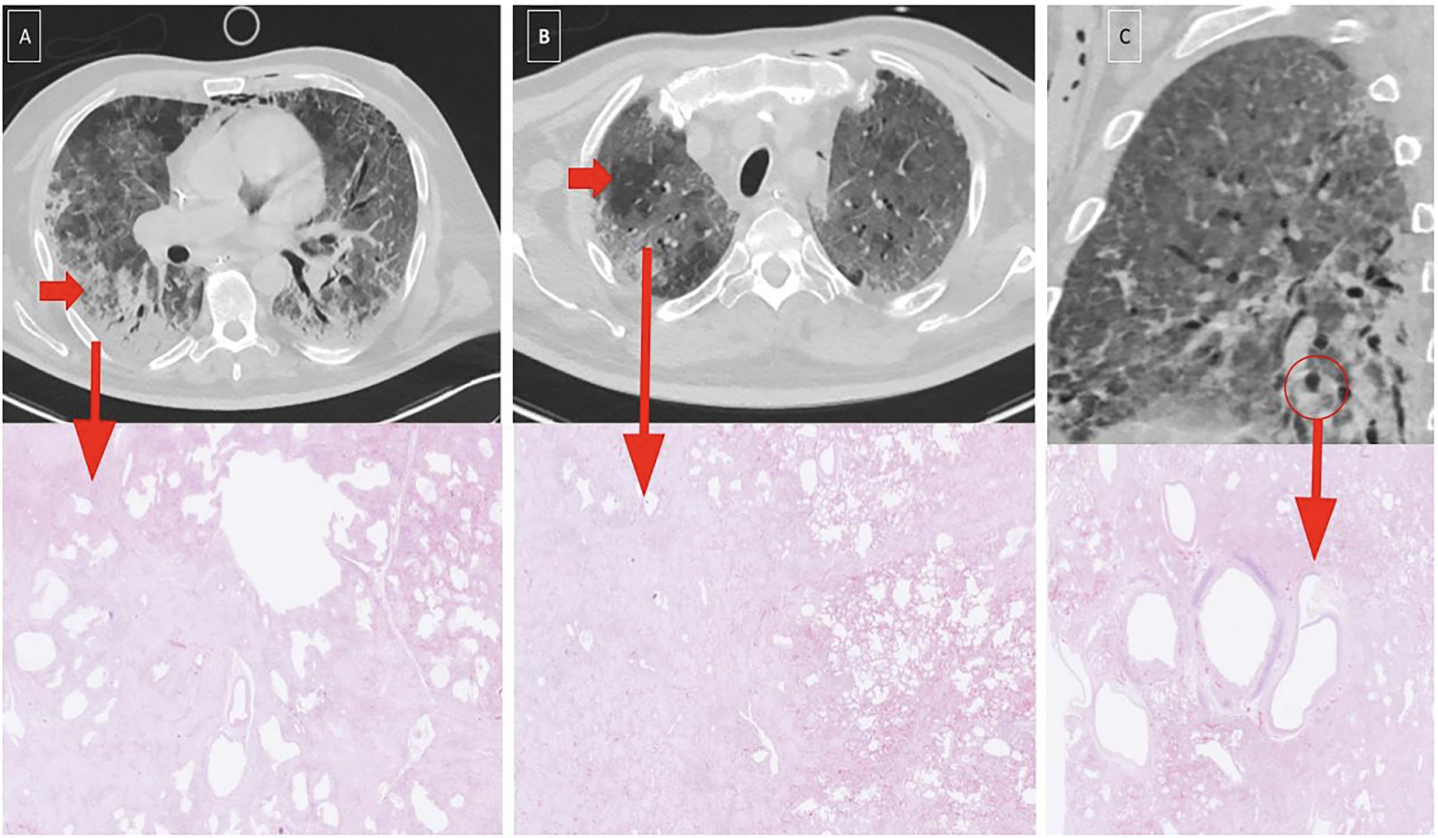
CT (day 39) with correlated biopsy (day 55) from patient 4. A 61-year-old male developed ARDS secondary to COVID-19 and was treated in the ICU. (a–c) Top: Chest CT performed on day 39 after symptom onset showed progression compared to earlier imaging, with increased extent and density of GGOs, interlobular septal thickening, intralobular lines (crazy paving), and signs of barotrauma. Bottom: Post-mortem histological examination on day 55 showed acute alveolar edema, fibrin deposition, and hyaline membranes, consistent with DAD. In addition, areas of chronic inflammation (lymphocytes and macrophages), desquamated epithelium, reactive type II pneumocytes, and intra-alveolar fibrin plugs were present, indicating early transition to OP. ARDS, acute respiratory distress syndrome; CT, computed tomography; DAD, diffuse alveolar damage; GGO, ground-glass opacity; ICU, intensive care unit; OP, organizing pneumonia.

**Fig. 6.**
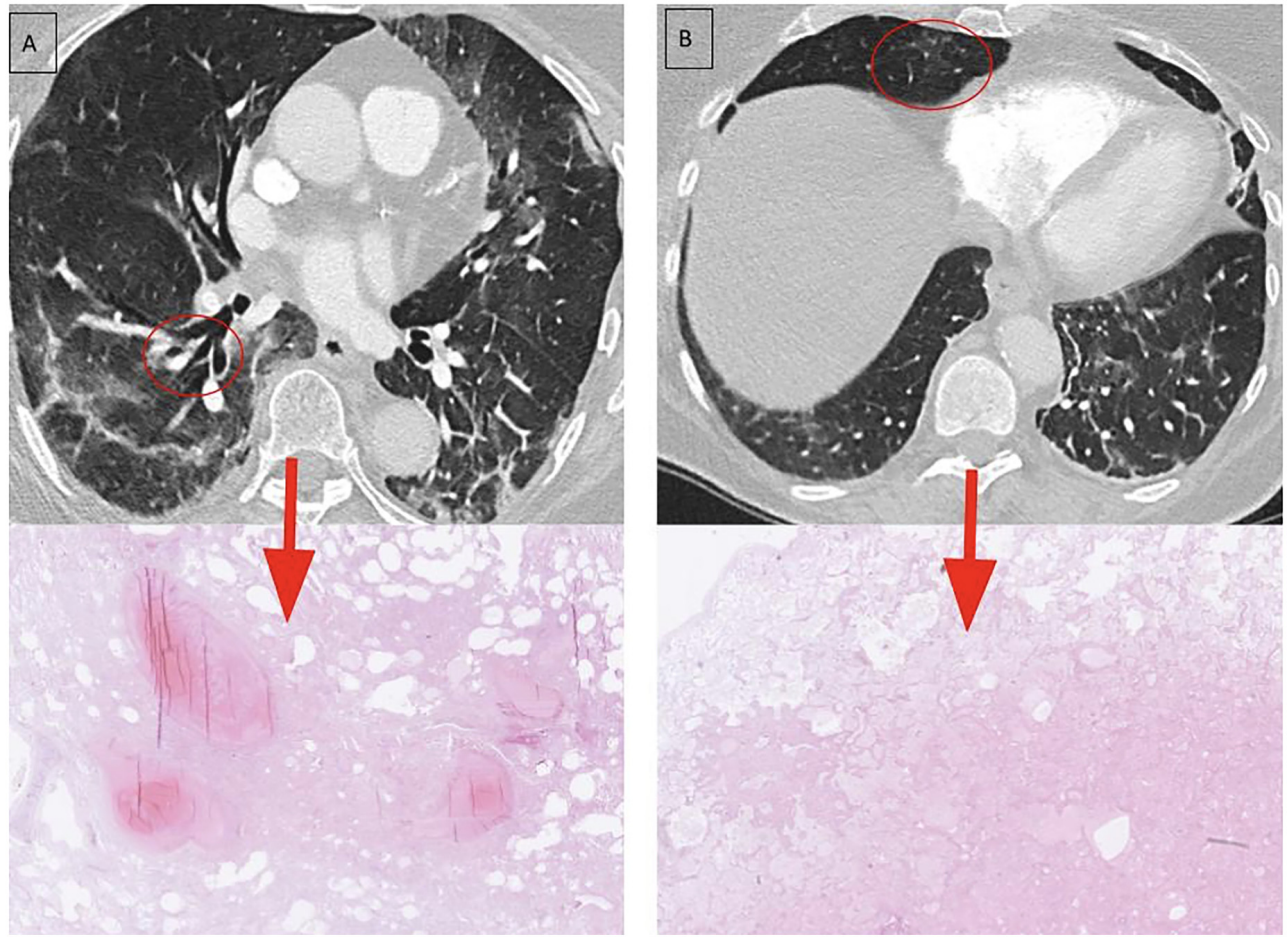
CT (day 11) with correlated biopsy (day 18) from patient 3. An 80-year-old woman presented with an 11-day history of shortness of breath. (a) CT performed on day 11 after symptom onset (top) showed GGOs and perilobular opacities, the latter frequently observed in OP at any stage. Linear consolidation parallel to the pleural surface was also noted, consistent with late phase of OP. Post-mortem histological examination on day 18 (bottom) revealed acute alveolar edema, fibrin deposition, intravascular fibrin thrombi, and features of OP. Notably, the patient had received anticoagulation (Tinzaparin) but nevertheless developed and died from pulmonary embolism on day 18 after symptom onset. CT, computed tomography; GGO, ground-glass opacity; OP, organizing pneumonia.

Examples of radiologic–pathologic correlation are illustrated in Figs. 5 and 6, showing two patients with matched CT images and corresponding post-mortem biopsy sites.

## Discussion

Our results confirm that CT patterns observed during the progression of COVID-19 correspond to post-mortem histopathological findings: GGOs matched alveolar edema, CPP reflected hyaline membranes (i.e. DAD), and OP was a frequent finding both radiologically and histologically.

Interestingly, thromboembolic events were identified histopathologically in half of the patients but were not detected on CT pulmonary angiography. The lack of correlation relating to thromboembolism could be attributed to the limited detectability of small peripheral emboli on CT. Such emboli are known to be common in COVID-19 (15) but may fall below the spatial resolution of CT imaging. Larger thromboemboli identified at autopsy were likely contributing factors to death and may not have been present at the time of imaging.

Our findings demonstrate a strong correlation between radiological and histopathological features of edema, DAD, and OP, which may be of particular importance in patients who show radiological deterioration and may therefore require intensified monitoring. Both imaging and histopathology contribute to understanding the pathophysiology of the disease and complement each other by providing partly distinct information reflecting a snapshot of disease status. Improved knowledge of the pulmonary progression of COVID-19 may help clinicians better determine a patient's stage within the disease course. Furthermore, we present eight patients in whom CT was performed within a few days of death, or on the same day, followed by post-mortem examination.

Our findings suggest that the CT pattern and the histopathological patterns do correspond. Furthermore, in our cases, there is a spatiotemporal correlation, i.e. in terms of space and time. However, our second aim, to determine whether an agreement and correlation could be done retrospectively, proved to be possible but time-consuming.

Radiology–pathology correlations reported in the literature for patients with ARDS indicate that, in acute severe COVID-19, GGOs, CPP, and airspace consolidations correlate with edema and inflammation whereas perilobular patterns and the reverse halo sign are associated with OP (20).

Edema in the alveolar compartments has been widely reported, which is typically the first sign visible in a light microscope. This edema occurs through increased permeability through the barrier of the capillary endothelium and alveolar epithelium in the alveolar septum and corresponds to the GGO image on CT. Focal fibrin precipitation is also seen in the alveolar spaces, in typically rounded formations forming fibrin plugs (21). Mononuclear cells (lymphocytes and monocytes/macrophages) have been reported focally in the fibrin (21). Elements of slight thickening of the alveolar septa with mild chronic inflammation also occur in the interstitium.

These findings are consistent with those of Morin et al., who reported similar histopathological features in a large prospective series of 169 patients who died from COVID-19-related ARDS (22). Although their study employed post-mortem percutaneous lung biopsies using 14-G needles, our approach relied on full autopsy samples, enabling broader anatomical correlation. Despite differences in methodology and sample size, the overlap in pathological features underscores the reproducibility of these findings across different patient populations and sampling techniques.

Another issue under investigation is the degree and type of developed fibrosis post COVID-19 infection (23). Given the phylogenetic relationship between SARS-CoV-1 and SARS-CoV-2 (24) it is conceivable that a proportion of survivors will develop progressive interstitial lung disease (ILD). ILD comprises a large heterogeneous group of pulmonary diseases characterized by inflammatory and/or fibrotic changes. ILD engages the connective tissues (the interstitium) and leads to inflammation and sometimes to different degrees of fibrosis in the lungs.

One histopathological pattern relevant in this context is OP, which represents a fibrotic healing process that does not necessarily always follow infectious lung injury. The ordinary course is gradual resorption of the loose fibrosis. However, failure of this process leads to collagen deposition and irreversible fibrosis, as seen in acute lung injury models (6,20).

We still lack complete knowledge about the course of the disease over time and which factors in the acute phase of the disease might be of particular importance for the long-term outcome. We hypothesize that the parenchymal changes that some COVID-19 patients develop might have much in common with OP. There are large knowledge gaps surrounding ILD and the initiated parenchymal inflammation. The treatments are therefore strongly limited. Histologically, the knowledge we possess today regarding COVID-19 pneumonia comes from studies performed on autopsy material from patients who have faced an acute phase of the disease (16,25) such as our patients described.

We also aimed to determine whether a spatiotemporal correlation could be established between radiological patterns and histopathological findings. We hypothesized that the radiologic features seen on CT would correspond to specific histological patterns, allowing imaging to predict post-mortem pathology. Establishing such a correlation could, in principle, enable radiologists to identify patients at risk of developing severe disease manifestations earlier in the clinical course and potentially guide more proactive management strategies. However, this type of correlation requires a robust methodology and larger cohort than was available in our study. A key limitation was the retrospective nature of the autopsy material. We were not able to retrospectively identify the precise anatomical origin of all tissue blocks with sufficient certainty to support detailed regional comparisons with CT.

Our study offers insights into the radiological–histopathological correlation in fatal COVID-19, but larger studies are needed for validation. Although imaging and pathology showed visual concordance, the lack of prospective sampling and timing inconsistencies may limit generalizability. The modest number of patients (n = 8) included presents a limitation of our study. However, the purpose of the study was descriptive radiologic–pathologic correlation rather than statistical inference. One important reason for the limited number of participants was that relatively few COVID-19 patients were autopsied during the study period at our hospital. Histological orientation challenges were mitigated by multiplanar reconstruction (MPR), though it was time-consuming. Due to the descriptive nature and absence of a control group, the findings should be interpreted cautiously.

In conclusion, our findings suggest that chest CT patterns in COVID-19 progression align with post-mortem lung histopathology. In the early phase, peripheral GGOs correlate with alveolar edema. After 1–2 weeks, GGOs become denser and evolve into CPP, corresponding to hyaline membranes (DAD). This correlation highlights CT's potential to identify the risk of ARDS, enabling earlier, targeted interventions. Our case series contributes to understanding the radiological–pathological spectrum of acute lung injury in severe COVID-19.
